# Evaluating Composite Sampling Methods of *Bacillus* Spores at Low Concentrations

**DOI:** 10.1371/journal.pone.0164582

**Published:** 2016-10-13

**Authors:** Becky M. Hess, Brett G. Amidan, Kevin K. Anderson, Janine R. Hutchison

**Affiliations:** National Security Directorate, Pacific Northwest National Laboratory, Richland, Washington, 99352, United States of America; Agricultural University of Athens, GREECE

## Abstract

Restoring all facility operations after the 2001 Amerithrax attacks took years to complete, highlighting the need to reduce remediation time. Some of the most time intensive tasks were environmental sampling and sample analyses. Composite sampling allows disparate samples to be combined, with only a single analysis needed, making it a promising method to reduce response times. We developed a statistical experimental design to test three different composite sampling methods: 1) single medium single pass composite (SM-SPC): a single cellulose sponge samples multiple coupons with a single pass across each coupon; 2) single medium multi-pass composite: a single cellulose sponge samples multiple coupons with multiple passes across each coupon (SM-MPC); and 3) multi-medium post-sample composite (MM-MPC): a single cellulose sponge samples a single surface, and then multiple sponges are combined during sample extraction. Five spore concentrations of *Bacillus atrophaeus* Nakamura spores were tested; concentrations ranged from 5 to 100 CFU/coupon (0.00775 to 0.155 CFU/cm^2^). Study variables included four clean surface materials (stainless steel, vinyl tile, ceramic tile, and painted dry wallboard) and three grime coated/dirty materials (stainless steel, vinyl tile, and ceramic tile). Analysis of variance for the clean study showed two significant factors: composite method (p< 0.0001) and coupon material (p = 0.0006). Recovery efficiency (RE) was higher overall using the MM-MPC method compared to the SM-SPC and SM-MPC methods. RE with the MM-MPC method for concentrations tested (10 to 100 CFU/coupon) was similar for ceramic tile, dry wall, and stainless steel for clean materials. RE was lowest for vinyl tile with both composite methods. Statistical tests for the dirty study showed RE was significantly higher for vinyl and stainless steel materials, but lower for ceramic tile. These results suggest post-sample compositing can be used to reduce sample analysis time when responding to a *Bacillus anthracis* contamination event of clean or dirty surfaces.

## Introduction

The process for decontaminating a facility after anthrax contamination incident can be described in five phases: (1) environmental sampling, (2) laboratory sample analysis, (3) hazard mapping and assessment based on laboratory results; (4) decontamination, and (5) decontamination sampling to validate decontamination efforts [1–3]. Determining the extent of the contamination (hazard mapping) and assessment (areas of high versus low levels of contamination) are critical in developing decontamination plans. Currently available reagents and methods are effective in decontaminating large areas within a matter of days [4–6], but sampling, analysis, and full restoration efforts requires months of effort. The Centers for Disease Control and Prevention (CDC) reported that over 125,000 environmental samples were taken after the 2001 anthrax attacks, and the restoration effort took over three months to complete for some facilities, and more than a year to restore the American Media, Inc. (AMI) building [1, 7]. Improving response time by reducing the sampling area is not realistic because the hazard assessment and hazard mapping would be compromised. However, the laboratory analysis phase is a significant bottleneck [3, 7, 8], and streamlining those efforts could improve response time. One way to reduce laboratory labor, time, and cost is to implement a composite sampling campaign [8].

Composite sampling is the process of combining samples or sampling over a larger area using a single sampling media to reduce the total number of samples and sampling analysis while maintaining confidence in hazard mapping and assessment [8]. This can reduce the number of lab analyses and the number of collected samples needed, thereby reducing the overall remediation timeline. Composite sampling methods have been used and extensively used for soil analysis and within the food industry [8]. A recent study by Tufts et al. evaluated the utility of composite sampling for recovery and analysis of *Bacillus atrophaeus* Nakamura (ATCC 9372) spores from clean surfaces [9]. High concentrations of spores (> 10^7^/surface material) were deposited via aerosol onto stainless steel coupons. Two composite-based collection approaches were used in this study: (1) CDC sponge wipe method [2] and (2) a modification of the CDC method where only one surface of the sponge was used to sample each coupon (single medium single pass composite). Tufts et al. concluded that there was no statistical difference between the two protocols tested, and the modified CDC approach can be implemented to improve sampling and analysis time and cost [9].

Recent work has highlighted the need to evaluate spore collection for low concentrations of *Bacillus anthracis* spores, or surrogates, to provide assurance of public safety during a low release event or post-decontamination [3, 9–13]. To address this need, we conducted a statistically designed sampling study for the recovery of *B*. *atrophaeus* spores by either discrete sampling or composite sampling. The studies were designed to investigate six experimental factors: (1) composite methodology; (2) number of locations to composite; (3) deposition location of spores; (4) surface material type; (5) number of spores deposited; and (6) comparing these experimental factors using clean versus grime coated coupons. The recovery efficiency is considered the response variable and is used to investigate the effects of the experimental factors. The results of this work were used to evaluate the effects of spore concentration, surface materials, and composite methodologies on false negative rate (FNR), recovery efficiency (RE), and limit of detection (LOD).

## Materials and Methods

### Bacterial Culture Conditions

*B*. *atrophaeus* Nakamura ATCC 9372 (BG) was purchased from American Type Culture Collection (Manassas, VA). Spores were prepared as described previously [14]. Overnight cultures for each strain were grown in tryptic soy broth (TSB; #286220; BD, Franklin Lakes, NJ) at 30°C prior to being diluted 1:100 in 1.6% nutrient broth with casein hydrolysate yeast extract (CCY) salts as previously described [15]. The culture was sporulated with shaking at 200 rpm for 7 days at 30°C and then harvested by centrifugation for 10 min at 10000 × *g* at 4°C. Pellets were resuspended in sterile water and stored at 4°C for 7 days (to enhance vegetative cell lysis), then washed three times in ultra-pure (18 Ω) sterile water. Spores were passed through a 41 μm filter to remove remaining cellular debris. Final spore suspensions were >95% phase bright. To reduce spore stock variability, three independent spore cultures were prepared and combined.

### Surface Materials

Eighteen gauge stainless steel sheets (316L) were cut to the desired coupon size of 25.4 by 25.4 cm (645.16 cm^2^). Armstrong Excelon Vinyl Composition Tile (#51830; Armstrong World Industries Inc., Lancaster, PA) and glazed porcelain floor and wall tile (UPC 737104028264) was purchased from Home Depot and cut to size. Drywall panels (Item # 11725 Gold Bond 3/8 inch x 4 feet x 8 feet) were purchased from Lowe’s Home Improvement, cut to size, and painted with two coats of white paint (Sherwin Williams ProClassic^®^ Interior Acrylic Latex Enamel).

Surface materials (except painted wallboard) were washed in a 1% solution of Liquinox^®^ (Alconox Inc., New York, NY), rinsed three times in deionized water, and then air dried. After washing, all coupons were sterilized by autoclaving on a dry cycle for 90 minutes in Chex-All^®^ Sterilization Pouches (Propper Manufacturing Company Inc., Long Island City, NY). Painted drywall was wiped with Dispatch^®^ Hospital Cleaner Disinfectant Towels (Clorox, 69150, Oakland, CA) two times followed by wiping with sodium thiosulfate to neutralize the bleach, and then air dried. Once the washing and sterilization processes were completed, the coupons are considered clean surfaces.

### Coupon Grime Coating

A grime mixture was applied to clean test coupons to mimic background dust. For these studies, coupons were coated with either grime alone or a grime containing a biological component. The entire coupon was coated with the indicated grime type in the text. For the test design, 50% of the coupons tested were coated with grime alone; the remaining coupons were coated with the grime containing a biological component.

The grime mixture was prepared by combining 94 grams of Arizona Road Dust (ISO 12103–1, A1 Ultrafine Test Dust, Analysis 5430, Powder Technology Inc., www.powdertechnologyinc.com) and 3 grams of metro grime (Bay Area Rapid Transport car grime, collected and provided by Staci Kane on September 23, 2014). This coupon coating is referred to as “grime” in the text.

The grime mixture (i.e., Arizona Road Dust and Metro Grime) containing an additional 3 grams of biological mixture was made by combining: Lycopodium powder, (Catalog No. 215B, Duke Scientific, Palo Alto, CA), 50% w/w; and 50% w/w Ragweed pollen, (Catalog No 214B, Duke Scientific). This coupon coating is referred to as “grime and biological” in the text.

For both types of coating material, the mixture was sterilized by autoclaving two times on a liquid 90 minute cycle. The grime coatings (12.5 grams) were resuspended in 1 liter of 70% denatured reagent alcohol (Ricca Chemical 2546705, Pocomoke City, MD) and mixed vigorously on a stir plate. To provide a uniform coating, stainless steel or ceramic coupons were pre-coated with a light spray of 70% alcohol. The grime/alcohol mixture was then applied by pipetting with a targeted grime mass of 250 mg/coupon (30 mL volume per coupon). Coupons were air dried overnight then stored in a coupon holder as to not disrupt the grime. The total mass of grime deposited was 230 mg ± 50 mg (data not shown).

### Coupon Inoculation

Clean coupons were inoculated following the cleaning process described above. Grime coated coupons were inoculated after grime coating as described above. Spore stock solutions were enumerated prior to testing by dilution series in phosphate buffered saline (#10010049; Invitrogen, Waltham, MA) containing 0.02% Tween-80 (#P4780; Sigma-Aldrich, St. Louis, MO), which is denoted PBS-T. The spore stock was diluted to an appropriate concentration in PBS-T buffer such that a 50 μL volume would contain the desired number of spores. The spores were deposited onto the coupon surface material as 20 droplets (0.05 mL volume per droplet) as previously reported [10]. The inoculated materials were dried without interruption for approximately two hours.

### Surface Sampling Methods

Cellulose sponge samplers pre-moistened with neutralizing buffer (Sponge-Stick, P/N SSL10NB; 3M, St. Paul, MN) were used to sample coupons. Five sampling methods were used: 1) single medium, single pass discrete (SP-Discrete); 2) single medium multi pass discrete (MP-Discrete); 3) single medium, single pass composite (SM-SPC); 4) single medium, multi-pass composite (SM-MPC); and 5) multi medium, multi-pass sample composite (MM-MPC).

The SP-Discrete samples were collected using a single pass of the sponge across the surface of one coupon [9]. The MP-Discrete samples were collected with a single medium (sponge wipe) from a single clean or dirty coupon using the CDC four pass procedure [2, 16]. Samples were collected using overlapping ‘S’ pattern with horizontal strokes, the sponge was turned and the same area was wiped again using vertical ‘S’ strokes, the sponge was then turned on the edge to wipe using diagonal ‘S’ strokes, and finally the tip of the sponge was used to sample the material perimeter.

**Fig 1:**
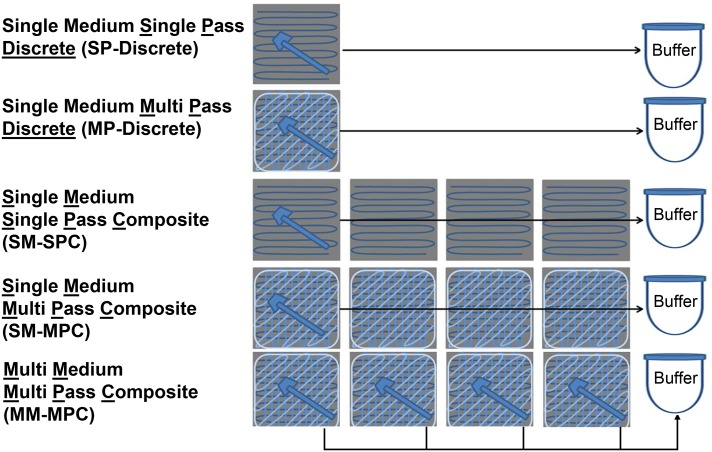
Pictorial overview of the sampling methods used in the study. Single medium single-pass discrete (SP-Discrete); single medium multi-pass discrete (MP-Discrete); single medium, single-pass composite (SM-SPC); single medium, multi-pass composite (SM-MPC); and multi-medium, multi-pass composite (MM-MPC) were used in the study. Buffer; Phosphate buffered saline containing 0.02% Tween 80.

Three types of composite methodologies were tested. The first method is a single medium, single pass composite (SM-SPC). In this method, a material (i.e., coupon) is sampled with a single pass of the sponge. A new face of this single sponge is used for each coupon sampled as reported by Tufts et al. [9]. The second methodology is the single medium, multi-pass composite (SM-MPC) where a single sampling medium is used to sample across multiple coupons using the CDC four pass procedure. The third methodology or the multi-medium, multi-pass sample composite (MM-MPC) uses a single sampling medium to sample a single coupon. Four, eight, or 16 coupons are sampled, each with a single sponge (Fig 1). Subsequently, an extraction step is conducted where one sponge is extracted at a time using one solution for all the sponges. Each sponge is removed from the stick and an extraction step is conducted in 90 mL of PBS-T buffer in a stomacher bag. Using the same buffer, each subsequent sponge (i.e., four, eight, or 16) undergoes the same extraction process in the 90 mL of buffer. The resulting buffer contains the extracted material from all locations that were designated to be composited (Fig 1).

### Sample Processing and Analysis

**Fig 2:**
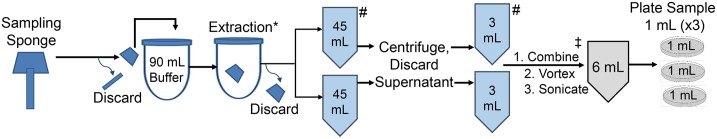
Pictorial overview of the sample process and analysis method for each sampling sponge. *For sponges used with the multi-medium multi-pass composite (MM-MPC) method, each of the sponges used for sampling were extracted one at a time, in the same 90 mL buffer. ^#^The volume following extraction was >90 mL (up to 105 mL) for MM-MPC samples; therefore, the sample was split into three aliquots prior to being combined. ^‡^The final target volume for all methods was 6 mL, including the MM-MPC method.

Using sterile technique, each test coupon was sampled with a 3M^™^ Sponge-Stick (3M, St. Paul, Minnesota; catalog number SSL10NB) as described in the Surface Sampling Methods section. The sponge was then transferred to a stomacher bag (Seward; catalog number BA6141). Ninety mL of PBS-T was added to the bag and the bag was processed in the stomacher for one minute at 260 rpm (Seward Stomacher 400 Circulator). Excess liquid was squeezed out of the sponge and then the sponge was discarded. The final elution was transferred to 50 mL conical tubes for centrifugation at 3,500 x *g* for 15 minutes. The supernatant was removed and the remaining solution (~3 mL each tube) was vortexed for 30 seconds and sonicated for 30 seconds two times. The two aliquots were pooled and the final volume was recorded. For the MM-MPC samples, the final volume exceeded a total of 90 mL, therefore, three aliquots were pooled following centrifugation [10]. From the total sample volume, 1 mL volumes were plated on tryptic soy agar (TSA) plates in triplicate (Fig 2) and incubated overnight; if there was no growth on the three plates a fourth sample was plated the following day (1 mL plated). The average value from the three plates was used to determine the total number of spores recovered (i.e., average CFU from plate counts multiplied by total sample volume). As described below, recovery efficiency was calculated by dividing this calculated number of spores recovered by the target CFU.

### Test Design

Experiments were conducted in two stages: clean coupons, then grime coated coupons. The clean study evaluated four different coupon materials: stainless steel, ceramic tile, vinyl tile, and painted drywall. For each experiment, four MP-Discrete samples were collected from each test material; these samples served as the reference positive controls for the composite methods being tested. Additional controls included a negative control coupon and a negative process control which consisted of a sponge wipe that was removed from the packaging and transferred into the stomacher bag. For each experiment 10 inoculation controls for the inoculum (i.e., number of spores deposited) were prepared by plating on tryptic soy agar (TSA) plates with the same spore suspension used to inoculate the coupons. A total of 5 spore concentrations were tested: 5 CFU/coupon (0.00775 CFU/cm^2^), 10 CFU/coupon (0.0155 CFU/cm^2^), 25 CFU/coupon (0.0388 CFU/cm^2^), 50 CFU/coupon (0.0775 CFU/cm^2^), and 100 CFU/coupon (0.155 CFU/cm^2^).

Two contamination deposition locations were tested: spores deposited across all of the surfaces or on one random sample (for 4, 8, and 16 coupon composite tests). To determine if there was a location effect, all coupons or only one randomly assigned coupon were inoculated.

The grime coated study evaluated three different coupon materials: stainless steel, ceramic tile, and vinyl tile. Spores were deposited after the grime coating was applied as described above. There were 4 MP-Discrete samples collected from each test material and concentration. Spores were either deposited across all of the coupons or on one random coupon (for both 4 and 8 coupon composite tests).

### Statistical Analysis

A plot of the theoretical versus sample quantiles (QQ-plot) of the residuals (theoretical quantiles versus sample quantiles) was used to confirm the data was normally distributed (S1 Fig) as previously described [17]. An analysis of variance was conducted to evaluate the difference among groups and the variability among different aspects of this experiment. Analysis of Variance, known as ANOVA, is a statistical methodology that analyzes for variability in the data set. Using ANOVA, hypothesis tests are conducted to test for differences between the groups based on their means. F statistics are used to determine if there are significant differences in the group analysis. These F statistics provide insight into whether a group is significant based on the ratio of variance between groups and within groups [18].

For analyses including both clean and grime coated samples, the split-plot nature of the data was taken into account when performing ANOVA; the CFU target factor was treated as a split plot restriction on randomization. Therefore, the number of CFU targets used is the determining factor for the degrees of freedom in the analysis (e.g., if four CFU targets are being compared, then the analysis would have three degrees of freedom). When performing ANOVA, all six experimental factors for all the test runs were analyzed together. The purpose of the statistical analysis is to determine the effects of the factors of interest; to account for the variability across the test matrix and determine the significance between levels of each factor on spore recovery efficiency. Significant interactions between independent experimental factors can also be discovered in this way, as described by Chambers et al. [18]. Data presented as box plots were constructed as previously described [19].

False negatives occur when contaminant is not detected from a coupon when the contaminant was known to have been deposited on the coupon. False negative rate (FNR) is the probability that contaminated samples in the experiment will actually be identified as negative for the contaminant. False negative rates were used to estimate the limit of detection at the 90 percent threshold (LOD_90_) as previously described [10, 12, 13] for the process method used in the studies. The LOD was calculated using linear interpolation between the FNR.

## Results

In these experiments, we varied six independent experimental factors (Table 1): (1) Composite Methodology; (2) Number of Locations to Composite; (3) Deposition Location of the Spores; (4) Surface Material Type; (5) Number of Spores Deposited; and (6) Presence of Grime. Recovery efficiency was considered the response variable and is used to investigate the effects of these factors. Recovery efficiency (RE) was computed using the number of colony forming units (CFU) for the sample divided by the target CFU.

**Table 1:** The six experimental factors used in the study to evaluate recovery efficiency.

Experimental Factor	Abbreviated Nomenclature, Long	Abbreviated Nomenclature, Short	Comparison	Comments
**Composite Methodology**	Composite Method	Composite	Single pass versus multiple passes across coupons; multi media versus single media	Refer to Figs 1 and 2 for a diagram of the sampling and processing methods
**Number of Locations to Composite**	Number of Locations	Locations	4 locations versus 8 locations	Separate tests with 16 locations composited were also performed using the MM-MPC method
**Deposition Location of the Spores**	Number Contaminated	Contaminated	Spores distributed across all the coupon locations versus spores randomly placed on only one coupon	Number contaminated refers to the number of coupons (i.e., 1 random coupon versus all coupons)
**Surface Material Type**	Coupon Material	Material	Four unique materials, all compared to one another	Ceramic Tile, Stainless Steel, Painted Drywall, and Vinyl Tile
**Number of Spores Deposited**	CFU Targets	CFU	Range of colony forming units (CFU)	Target CFU of 5, 10, 25, 50, and 100 CFU per coupon
**Coupon Coating**	Presence of Grime	Grime	Clean coupons versus coupons coated with grime materials.	Coupons surfaces were clean, coated with grime alone, or coated with grime containing the biological component (refer to Materials and Methods)

### Comparing SM-SPC and SM-MPC methods for RE

The first set of experiments used clean coupons to compare the single medium single pass composite (SM-SPC) method against the single medium multi pass composite (SM-MPC) method at the highest tested inoculum, 100 CFU/coupon, and either 4 or 8 coupons were inoculated. Two contamination deposition locations were tested: spores deposited across all of the coupons (i.e., all locations) or on one coupon (i.e., one location) for both 4 and 8 coupon composite tests. As shown in Fig 1, both methods use a single sponge for multiple coupons; however, the difference between these methods is the number of passes used to sample each coupon. The single pass method uses only a single pass per coupon (as described by Tufts et al. [9]) whereas the multi pass method uses multiple passes across each coupon sampled as described by the CDC [2].

**Fig 3:**
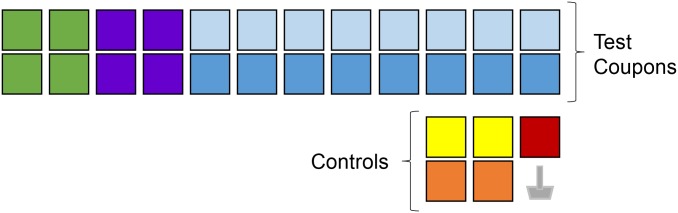
Pictorial overview of the test design used to compare the single media single pass composite (SM-SPC) method with the single media multi pass composite (SM-MPC) method. Each method was tested using 4 or 8 locations composited. On one test day, all locations for all tests were contaminated. On the other test day, only one random location was contaminated. Gray Sponge: Negative Process Control. Each square represents an individual coupon location. Green: SM-SPC method with 4 coupons; Purple: SM-MPC method with 4 coupons; Light Blue: SM-SPC method with 8 coupons; Dark Blue: SM-MPC method with 8 coupons; Yellow: SP-Discrete serving as process control for the SM-SPC; Orange: MP-Discrete serving as process control for the SM-MPC; Red: Negative control coupon.

The experiments were conducted using two different runs, one run with all locations contaminated and the other with only one random location contaminated. For each run, a total of 29 coupons for each coupon material were used and one negative process control was used, for a total of 30 samples as shown in Fig 3. In total, eight runs were completed (two runs for each material, four materials). The raw data from these experiments are provided in S1 Table.

A two sample paired t-test was performed to determine if there was a statistical difference in RE for the SM-SPC method versus the SM-MPC when all the independent experimental factors are considered. The t-test results showed a significant difference in RE for the SM-SPC method versus the SM-MPC method (p = 0.0063) at the 100 CFU/coupon target. The RE mean for the SM-SPC method was significantly lower than the RE mean for the SM-MPC method for all coupon materials when 4 or 8 locations were composited. As stated previously, tests were conducted at the highest investigated target CFU to compare these two methods, but it should be noted that this is orders of magnitude lower CFU numbers than have often been used to investigate spore RE from materials using swabbing methods (>10^7^ spores per surface) [3, 9]. Because the remaining CFU target numbers for the full factorial study are less than 100 CFU/coupon (expected to be associated with lower RE), the SM-SPC method was eliminated from continued testing with lower CFU targets.

### Testing clean coupon surfaces, full factorial study

The test matrices for the full factorial composite study are provided in S2 Table (Matrices 1 through 40). The raw data from these experiments (160 runs with 20 positive process controls) are provided in S3 Table. The test design was consistent with the tests comparing SM-SPC with SM-MPC methods with one exception: for each test run, there were four positive controls run using the MP-Discrete method (versus two using SP-Discrete and two using MP-Discrete). The tests were conducted using 4, 8, or 16 locations. For each coupon material and target CFU, there were two test runs completed: on one test day, all coupons (i.e., all locations) were contaminated; on the other test day, only one random coupon (i.e., one location) was contaminated.

Two different statistical analyses were performed on these data. One analysis was performed to determine the significance of each of the experimental factors on the response variable, recovery efficiency (RE), including all tests done with 8 or fewer locations for the composites. The second analysis performed included the 16 sample location composites and was specifically focused on the significance of the number of locations (4, 8, or 16) for composite sampling. For this analysis, we included data from experiments conducted using four additional test matrices (S2 Table, matrices 41 through 44).

#### ANOVA results investigating experimental factors and their interactions

ANOVA was performed in order to compare the differences of the experimental factors (Table 2) and their interactions. Data from experimental runs with a 5 CFU target resulted in recovery of no bacteria (CFU) in 53.1% of the experimental runs (S3 Table). As a result, the data obtained for the 5 CFU target was omitted from these analyses unless otherwise specified. With the 5 CFU target concentration data removed, the normal distribution assumption was satisfied for standard ANOVA as described in Materials and Methods. The ANOVA analysis was performed using all the data generated from the full factorial study with four CFU targets (10, 25, 50, 100 CFU). The results of the ANOVA are provided in Table 2.

**Table 2:** ANOVA results showing statistical significance for the tested factors and their interactions for clean coupon materials.

Source	DoF^a^	SumSq^b^	MeanSq^c^	F-value	P-value
**CFU Target**	3	0.8473	0.2824		
**Coupon Material**	3	0.7015	0.2338	6.3811	0.0006
**Composite Method**	1	1.6618	1.6618	45.3485	< 0.0001
**Number Contaminated**	1	0.0544	0.0544	1.484	0.2264
**Number of Locations**	1	0.1057	0.1057	2.8852	0.0929
**CFU: Material**	9	0.6138	0.0682	1.8611	0.0684
**CFU: Composite**	3	0.0158	0.0053	0.144	0.9333
**Material: Composite**	3	0.1758	0.0586	1.5987	0.1954
**CFU: Contaminated**	3	0.3521	0.1174	3.2024	0.0271
**Material: Contaminated**	3	0.1384	0.0461	1.2593	0.2933
**Composite: Contaminated**	1	0.0109	0.0109	0.2974	0.5869
**CFU: Locations**	3	0.0599	0.02	0.5447	0.6530
**Material: Locations**	3	0.0216	0.0072	0.1968	0.8984
**Composite: Locations**	1	0.0722	0.0722	1.9712	0.1638
**Contaminated: Locations**	1	0.0034	0.0034	0.092	0.7624
**Error**	88	3.2249	0.0366		
**Total (corrected)**	127	8.0569			

^a^Degrees of freedom

^b^Sum of squares

^c^Squared mean value

Based on the ANOVA results, we found that two of the independent experimental factors were significant with a p-value less than 0.05 (Table 2): surface material (p = 0.0006) and composite method (p < 0.0001). The surface materials were selected in order to give a range of recovery efficiencies, so this significant difference was expected. Ceramic tile had the highest RE (41%), followed by stainless steel (37%) and drywall (29%); vinyl tile had the lowest RE (24%).

**Fig 4:**
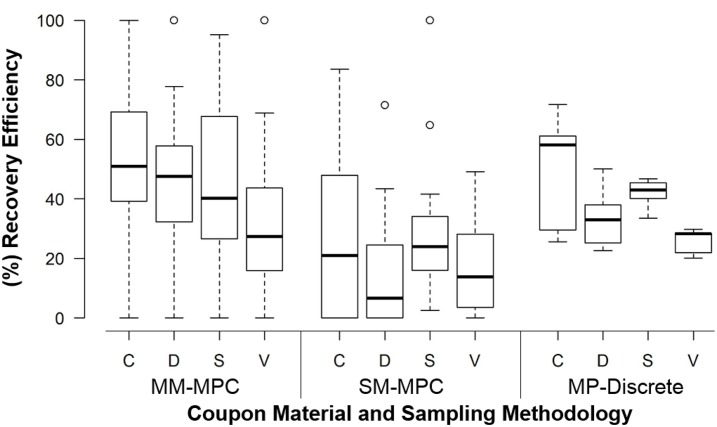
Coupon material and compositing methodology are significant experimental factors on the response variable, recovery efficiency. Of the materials tested, ceramic tile and stainless steel had the highest recovery efficiency and vinyl tile had the lowest RE. For all materials and CFU targets tested (10, 25, 50, 100 CFU), the MM-MPC method yielded higher RE than the SM-MPC method. The process controls, MP-Discrete, data are included for comparative purposes. C = ceramic; D = drywall; S = stainless steel; V = vinyl tile.

Additional analyses were conducted to determine if the MM-MPC method had the highest RE, and if this RE was significant compared to the other composite methods used. As shown in Fig 4, the RE was highest for all coupon materials using the MM-MPC method compared to the SM-MPC method, and this difference is significant. Regardless of the compositing method, vinyl and drywall had the lowest RE of the tested coupon materials. However, the material with the greatest magnitude difference in RE using the different composite methods was drywall. Using the MM-MPC method, the median RE for drywall was 50% compared to ~10% using the SM-MPC method. These results suggest that RE can be improved using the MM-MPC method over the SM-MPC method even with surface materials that are associated with low RE.

#### The MM-MPC method yields higher RE than SM-MPC sampling method when four or eight locations are composited

**Fig 5:**
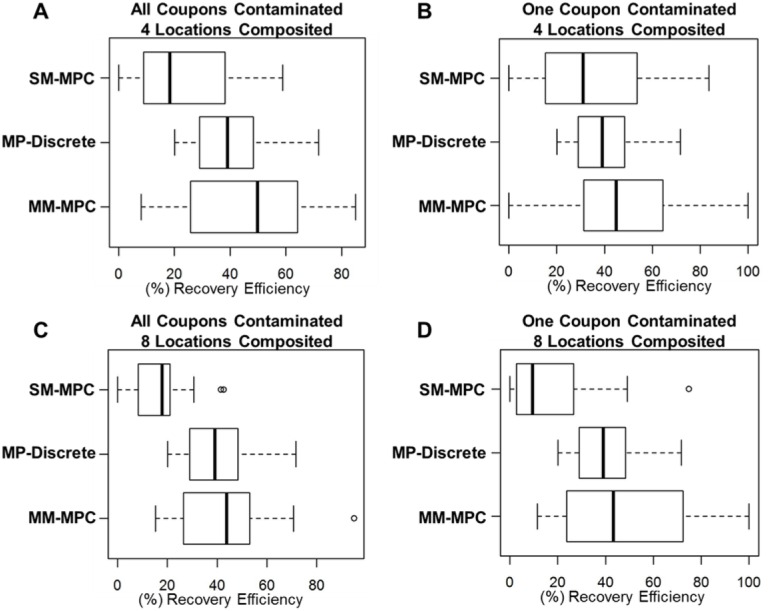
The composite methodology is significant, and the MM-MPC method yields the highest RE when considering the number of locations composited and number of coupons contaminated. The four scenarios tested were 4 samples composited with (A) all or (B) one random coupon contaminated and 8 samples composited with (C) all or (D) one random coupon contaminated. The process control, MP-Discrete, data is included for comparative purposed for the composite methods.

As shown in Fig 5, the MM-MPC method had a consistently higher RE than the SM-MPC method. This was confirmed by the ANOVA, where there was a significant difference between the methods (p-value < 0.0001). This was the case whether all coupons were contaminated or just one, as well as if 4 samples were composited or 8 samples composited. In each of those cases, the MM-MPC method had RE values that were slightly higher than MP-Discrete samples. This shows that combining samples, especially after sampling (MM-MPC) is as good as using the discrete sampling approach (MP-Discrete). Although the same number of sponges must be extracted using the MM-MPC as the MP-Discrete method, the number of samples to be analyzed at the end point of the method (refer to Fig 2) is reduced.

#### The two-way interaction between CFU target and the number of contamination locations is significant

**Fig 6:**
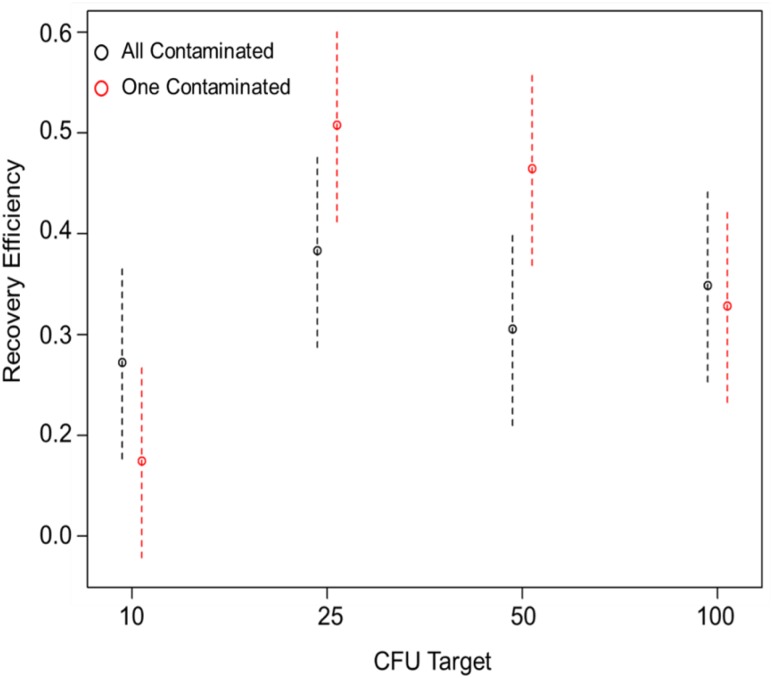
The interaction of two experimental factors, CFU target and number of contaminated locations, significantly affect recovery efficiency (p = 0.0271). The means are plotted as open circles and the error bars are representative of two standard deviations.

As shown in Table 2, the only two-way interaction that was statistically significant (p < 0.05) was between the CFU Targets and the number of contamination locations (one random coupon versus all coupons contaminated) (p-value = 0.0256). This interaction is shown in Fig 6. At 10 CFU target per coupon, the all coupons contaminated composite samples had a higher RE than those composite samples with only one coupon contaminated. However, at 25 CFU and 50 CFU target per coupon, the composite samples with only one coupon contaminated had a higher RE than those with all coupons contaminated. At 100 CFU target per coupon, both had similar RE values. The random order of the one coupon contaminated samples could be responsible for this; however this study was not designed to determine the effect of the random coupon swipe order. Therefore, no further testing was done to investigate this interaction.

#### Increasing the number of locations composited after sampling does not reduce RE

For this study, we focused on the comparison of the number of locations to composite after sampling: 4 individual locations, 8 individual locations, or 16 individual locations at one time. The data set used for these results was obtained from a collaboration of test matrices 41–44 and a subset of the data from matrices 1–40 (S2 Table).

We obtained a balanced data set with 24 values where an analysis of 4, 8, and 16 locations could be compared. To obtain this balanced set of data, the data set was restricted to two CFU target values (10 and 100 CFU), two types of coupon materials (ceramic tile and stainless steel), and sampling only with the MM-MPC method. Both deposition locations of contamination (all coupons locations or only one random coupon location) were tested with this set of data. Using this subset of data, an ANOVA was conducted as previously described with four factors and the results are shown in Table 3.

**Table 3:** ANOVA results for experiments with 16 locations composited using the MM-MPC method.

Source	DoF^a^	SumSq^b^	MeanSq^c^	F-value	P-value
**CFU Targets**	1	0.0008	0.0008		
**Coupon Material**	1	0.2067	0.2067	2.5649	0.1437
**Number Contaminated**	1	0.0458	0.0458	0.5688	0.4700
**Number of Locations**	2	0.0527	0.0264	0.3273	0.7291
**CFU: Material**	1	0.0005	0.0005	0.0059	0.9402
**CFU: Contaminated**	1	0.049	0.049	0.6085	0.4554
**Material: Contaminated**	1	0.0867	0.0867	1.0761	0.3266
**CFU: Locations**	2	0.0424	0.0212	0.263	0.7745
**Material: Locations**	2	0.0773	0.0386	0.4796	0.6341
**Contaminated: Locations**	2	0.0615	0.0308	0.3818	0.6932
**Error**	9	0.7252	0.0806		
**Total (corrected)**	23	1.3487			

^a^Degrees of freedom

^b^Sum of squares

^c^Squared mean value

**Fig 7:**
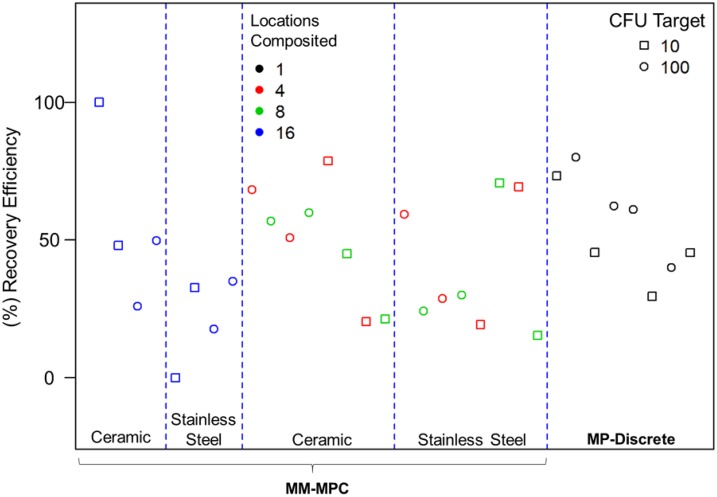
Increasing the number of locations composited from 8 to 16 does not reduce RE. The experiments were conducted using the MM-MPC methodology with 1, 4, 8, or 16 locations composited. The data for ceramic and stainless steel coupons are provided together in the MP-Discrete data set.

The results of the ANOVA revealed zero significant F tests for any independent experimental factor or any two way interactions. As shown in Fig 7, in one case (100 CFU on ceramic), RE was higher when 16 locations were composited. However, for all other tests, RE was neither improved nor reduced when 4, 8, or 16 locations were composited, and RE were similar to those obtained using the MP-Discrete method. These data indicate that increasing the number of locations composited to 16 locations using the MM-MPC method does not adversely affect RE.

#### ANOVA results investigating experimental factors and their interactions on grime coated coupons

We conducted tests with grime coated coupons using only the MM-MPC method. Spores were either deposited across all of the locations or on one random location for the 4 or 8 locations composite at three of the five target CFU concentrations tested for the clean samples (10, 25, and 50 CFU/coupon). The test design described for the full factorial study with clean coupons was recapitulated with the grime coated coupons. The raw data from these experiments are provided in S4 Table.

An ANOVA was performed in order to compare the differences of the experimental factors and their variations. The results of the ANOVA are provided in Table 4. Based on the ANOVA results, we determined that the main experimental factor influencing recovery efficiency was the presence of grime on the coupon surface (p < 0.05). In addition, we found that the interaction between the coupon material and grime presence was also significant (p = 0.0017).

**Table 4:** ANOVA results show that the presence of grime and the interaction between coupon type and grime presence are significant for RE.

Source	DoF^a^	SumSq^b^	MeanSq^c^	F-value	P-value
**CFU Targets**	2	0.2207	0.1103	NA	NA
**Coupon Material**	2	0.0323	0.0161	0.3082	0.7356
**Number Contaminated**	1	0.0253	0.0253	0.484	0.4886
**Presence of Grime**	2	0.3456	0.1728	3.3002	0.0418
**CFU:Material**	4	0.2724	0.0681	1.3009	0.2766
**CFU:Contaminated**	2	0.2712	0.1356	2.5896	0.0812
**Material:Contaminated**	2	0.0926	0.0463	0.8846	0.4168
**CFU:Grime**	4	0.4813	0.1203	2.298	0.0658
**Materal:Grime**	4	0.9916	0.2479	4.7347	0.0017
**CFU:Grime**	2	0.1893	0.0947	1.8079	0.1705
**Error**	82	4.2934	0.0524	NA	NA
**Total (corrected)**	107	7.2157			

^a^Degrees of freedom

^b^Sum of squares

^c^Squared mean value

**Fig 8:**
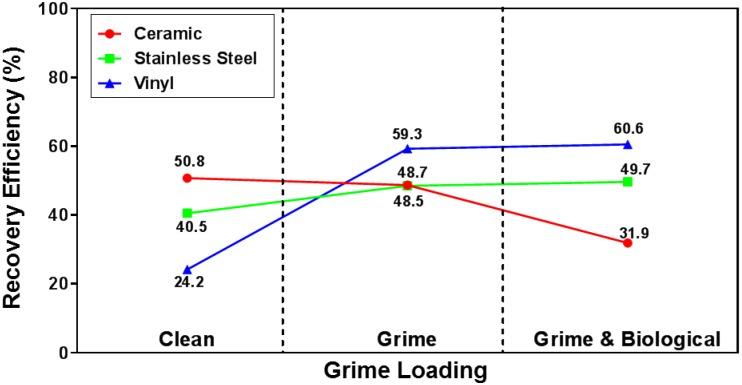
Grime coating on coupons is associated with an increased RE for stainless steel and vinyl coupons, but RE is reduced when grime is present on ceramic coupons. The presence of grime is an independent significant factor on RE. The interaction between material type and grime presence is also significant for RE.

The significant interaction between material and grime presence (Table 4) is plotted in Fig 8. The vinyl material had the broadest range of recovery efficiency values, and the RE for grime coated vinyl surfaces was 35% higher than the clean vinyl surface. Although there wasn’t a difference in RE between ceramic and stainless steel surfaces when the surfaces contain grime, there were significant differences in RE between these materials on clean surfaces and grime and biological surfaces. In the case of clean surfaces, ceramic had higher RE values by 10%, but in the case of grime and biological surfaces, stainless steel had higher RE values by almost 10%. This inconsistency, as shown by the intersecting lines in Fig 8, is why the interaction was significant.

### False negative rates (FNR) and limit of detection (LOD) with clean and grime coated coupons

**Fig 9:**
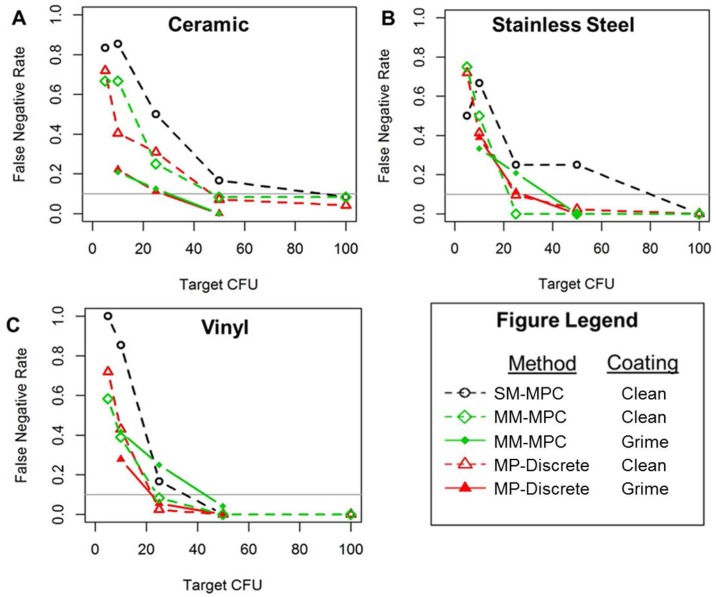
The FNR for the MM-MPC and MP-Discrete methods are similar for grime coated and clean coupons. The highest FNR were associated with the SM-MPC method. FNR decreases as CFU increases, and is consistently lowest at 100 CFU for ceramic (A), stainless steel (B), and vinyl (C) surfaces that are either clean or grime coated. The gray line in each plot demarcates the 0.1 FNR threshold (i.e., LOD_90_). The CFU targets used were 5, 10, 25, 50, and 100 CFU per coupon. Coupons were contaminated in four or eight locations for the SM-MPC and MM-MPC methods.

The FNR for the different coupon materials tested (stainless steel, vinyl, and ceramic) are shown in Fig 9. We observed that the FNR increased as the target CFU decreased, with the highest FNR at 5 or 10 CFU for all materials and sampling methods tested. However, with the exception of stainless steel coupons at the 5 CFU target, the SM-MPC method had the highest FNR for all coupon types and CFU targets of 10 or higher. The FNR for the MM-MPC method are similar to those obtained using the MP-Discrete method for clean and grime coated coupons for all CFU targets tested. These data indicate that false negative rates do not increase when using the MM-MPC method compared to the MP-Discrete method.

**Fig 10:**
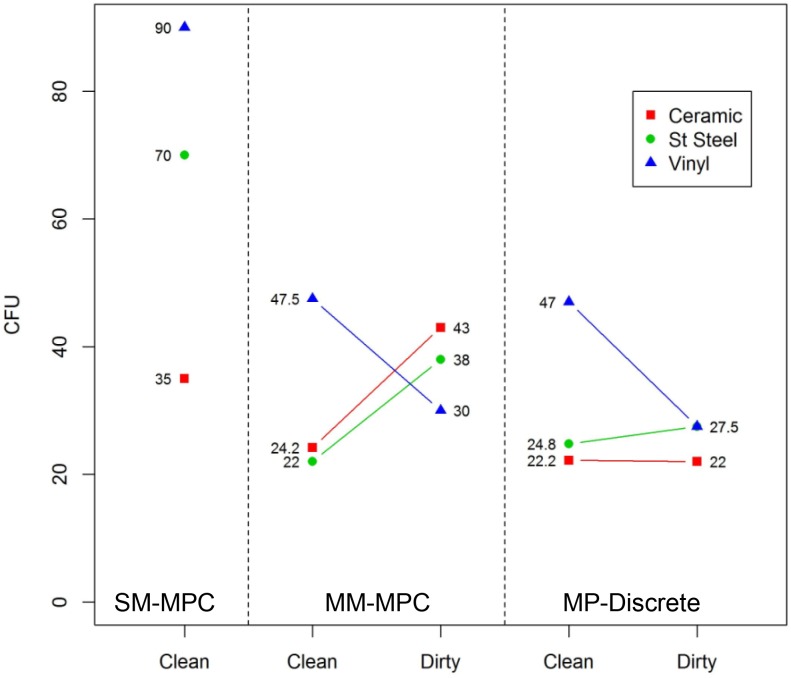
Limit of Detection (LOD90) is lower using the MM-MPC method for each coupon material compared to the SM-MPC method. The CFU targets used were 5, 10, 25, 50, and 100 CFU per coupon. Coupons were contaminated in four or eight locations for the both composite methods.

The limit of detection (LOD) at the 0.1 threshold (LOD_90_) is shown as the gray line in each plot in Fig 10. LOD_90_ is calculated by using a value of 0.10 for FNR to determine the CFU value using interpolation. These values are shown in Fig 7. The SM-MPC method was only used on clean surfaces and the LOD_90_ values for the SM-MPC method are significantly higher than LOD_90_ values using the MM-MPC method or MP-Discrete samples for clean surfaces.

As shown in Fig 10, the LOD_90_ for clean coupons are similar between the MM-MPC and MP-Discrete methods and on vinyl coupons coated with grime. However, on grime coated ceramic coupons, the MP-Discrete method had lower LOD_90_ values compared to the MM-MPC method. These results are consistent with reduced RE for ceramic coupons coated with grime (Fig 8). Taken together, these data (Figs 9 and 10) demonstrate that the FNR and LOD_90_ are lower for the MM-MPC than the SM-SPC methodology. Additionally, the MM-MPC method results are more consistent with the FNR and LOD_90_ when compared to MP-Discrete method.

## Discussion

We compared the single pass discrete (SP-Discrete) and multi pass discrete (MP-Discrete) sampling methods to three types of composite methodologies: 1) single medium, single pass composite (SM-SPC); 2) the single medium, multi-pass composite (SM-MPC); and 3) the multi-medium, post-sample composite (MM-MPC) (Figs 1, 2 and 3). Using this sampling approach, we investigated the effects of composite methodology, number of locations composited, deposition location of the spores, coupon material, and number of spores deposited on recovery efficiency (Table 1).

We applied ANOVA to our experimental results in order to compare the significance of these experimental factors and their interactions. We identified two significant factors and one significant interaction from the analysis (Table 2). The first significant experimental factor for recovery efficiency (RE) was the surface material (p = 0.0008) (Table 2). The coupon materials with the highest RE were ceramic tile (41%) and stainless steel (37%). The coupon materials with lower RE were painted drywall (29%) and vinyl tile (24%). Our RE are consistent with findings from different groups that have conducted spore recovery studies from different materials in that nonporous surfaces have been reported to have higher RE compared to painted or rough surfaces. Probst et al. reported reduced RE from rougher surfaces (13%) compared to nonporous, clean surfaces [20]. RE as high as 50% to 80% have been reported for clean stainless steel coupons; however, in those studies spore concentrations between 1x10^6^ to 2x10^7^ spores per coupon were used [9, 21–24] in comparison to our work which focused on lower concentrations of spores. As the number of spores used to contaminate the coupons increases, so does the RE; therefore, this difference between our work and reports by others was expected.

The second significant experimental factor on RE was the composite sampling method used (p-value < 0.0001) (Table 2). For all four coupon materials, the RE was significantly reduced when the single-medium multi-pass composite (SM-MPC) sampling method was used compared to both the MP-Discrete and the multi-medium post-sample composite (MM-MPC) sampling methods (Figs 1 and 2). These results indicate that in situations where the spore concentration is unknown, and the hot spots could contain low spore numbers, the MM-MPC method could be considered as a candidate method for sampling to reduce the number of analyses without sacrificing RE achieved with MP-Discrete samples. This is because the MP-Discrete method requires each sponge to be extracted in 90 mL of buffer. Thus, for 16 coupons, there would be 16x90 mL solutions to process post extraction using the MP-Discrete method. With the MM-MPC method, the RE is not significantly different than the MP-Discrete method and reduces the final sample handling to 1x~90 mL (up to 105 mL) solution for up to 16 coupons. For example, instead of centrifuging 32 to 48 sample tubes (16 coupons x 2 to 3 tubes per coupon) generated for 16 coupons with the MP-Discrete method, only 3 tubes are centrifuged and processed for the same number of coupons with the MM-MPC method. In addition, rather than 48 plate counts for 16 coupons with the MP-Discrete method, only 3 plate counts are required for the same number of coupons sampled with the MM-MPC method. The reduction in number of tubes centrifuged, number of tubes combined into a single sample, number of samples plated on agar, and number of plates requiring counting saves a significant amount of time, labor, and resources.

Our results are in agreement with the France et al. study conducted with Arizona test dust samples with medium spore concentrations (>1000 spores per sample). In their study, it was reported that the composite method was significantly different. However, their test materials (contaminated dust) vary greatly from coupon surfaces [3]; therefore, the relative agreement between our study and the France et al. study would need to be further assessed with additional testing of composite methods with environmental sample types and lower spore numbers.

The MM-MPC and SM-MPC methods with respect to the deposition locations of the spores and the number of locations composited were also compared. We tested four different scenarios: 4 or 8 locations composited with only one or all coupons contaminated. The MP-Discrete method was conducted in parallel for comparative purposes. In all four scenarios, the SM-MPC method resulted in significantly lower RE than the MM-MPC method and the MP-Discrete method. With the MP-Discrete method, there was little variance across all four scenarios tested, with a RE of ~30% (Figs 4 and 5). A consistent RE for the MP-Discrete method is expected based on previous work we have conducted as well as reports by others, which further bolsters these experimental results [5, 11–13, 21, 25]. These data also show that the median RE for the MM-MPC method is higher than the median RE for the MP-Discrete method, lending further support for using this compositing method rather than the SM-SPC method.

Previous work by Tufts et al. showed that the SM-SPC method resulted in a consistent spore RE from surfaces compared to a SM-MPC method at high target CFU (2 x10^7^) aerosolized spores deposited on stainless steel test coupons. Spores were deposited on only one coupon location, but the location of the contaminated coupon in the test varied (location one or location 4, with 4 locations composited). The results from the Tufts study indicated that most of the surface bound spores were recovered by the sponge in the first pass across the coupon surface, and therefore multiple passes could potentially be unnecessary [9]. Our results showed that both the single pass (SP-Discrete and SM-SPC) methods had reduced RE compared to all the multiple pass (MP-Discrete, SM-MPC, and MM-MPC) methods. However, this difference could be attributed to the lower spore concentrations used in our study compared to high concentrations used in the Tufts study, or could be attributed to spore application method (wet vs. dry aerosolized). In addition, the Tufts study only examined stainless steel coupons. In this study, stainless steel coupons as well as the other materials (ceramic tile, vinyl, and drywall) which have varying RE associated with these surface types were used. In addition, the data from this study was analyzed using ANOVA which compared all CFU target values with multiple surfaces associated with lower RE. The data from our study suggests that multiple passes across the coupon surface would be required in order to maximize spore recovery.

In addition to the independent experimental factors that were significant for RE, we also identified that the two-way interaction between the CFU target and the number of coupons contaminated is significant (p = 0.0271) (Table 2 and Fig 4). The results indicate that the difference between all or one coupon being contaminated varies with the CFU target. However, a consistent trend between CFU target and number of coupons contaminated is not readily apparent. At the lowest CFU target (10 CFU), the RE was 10% higher when all coupons were contaminated. At 25 and 50 CFU targets, the RE was also 10% higher, but for one coupon contaminated. At 100 CFU, the RE varied by only 2%, but was higher when all coupons were contaminated. We postulate that the random order of the contaminated coupon is a contributing factor for this variation at the lowest CFU target tested. However, this study was not designed to determine the effect of the random coupon swipe order on RE. The study conducted by Tufts et al. did vary the location of the contaminated coupon in their test design, and did report that the location of the coupon affected RE, but the variations between our test design and experiments conducted by Tufts et al. do not enable a direct comparison of these results [9]. Future studies are needed to determine the impact of contamination at different locations.

Based on the increased RE with MM-MPC compared to the SM-MPC method, we chose to further investigate the MM-MPC method with increasing numbers of locations composited. The purpose of these experiments was to determine if doubling the number of locations composited from eight to 16 would cause a reduction in the RE from ceramic or stainless steel coupons. We used 10 or 100 as the CFU target and compared these data to data sets from four and eight samples composited at the same CFU targets. ANOVA results from the experimental data did not show any significant differences between four, eight, or 16 individual locations composited. We found that the RE did not change as the number of locations composited increased (Table 3 and Fig 7), and there were no significant F tests (p < 0.05) for any independent experimental factor or two-way interaction. These results are similar to those reported by France et al. in which increasing numbers of samples composited did not significantly reduce the spore RE [3]. Taken together, these data suggest that compositing up to 16 samples using the MM-MPC method with a low spore concentration would not vary significantly from the results achieved using the MP-Discrete method. As stated previously, there is significant savings with respect to time and resources when using the MM-MPC method compared to the MP-Discrete method, and accuracy of RE is not sacrificed.

We also investigated the effect of grime coating on spore RE using ceramic, stainless steel, and vinyl coupons. In these experiments, we focused on the experimental factors that were significant: coupon material, composite method, and the two-way interaction of CFU target number and number of contamination locations. We conducted tests using the MM-MPC method and deposited spores on one coupon or across all coupons. The CFU targets tested in these experiments were 10, 25, and 50 CFU per coupon. When comparing data from these experiments to those done with clean coupons, we only compared data sets using the same CFU target numbers (i.e., 10, 25, and 50 CFU). We conducted tests with spores deposited on either one or all coupons. ANOVA results from these experiments showed that the presence of grime alone and the interaction between coupon material and grime presence (p = 0.0418 and p = 0.0017, respectively) were both significant factors for RE (Table 4 and Fig 8). Interestingly, the RE from ceramic tiles decreased when grime was present (31.9% versus 50.8%, respectively), but RE increased dramatically for vinyl coupons when grime was present (60.6% versus 24.2%, respectively). We postulate that this change in RE is due to unknown features of the ceramic surface, and not due to a biological effect from the grime on the *Bacillus* spores. This idea is further supported by the ANOVA results which show that the interaction between the presence of grime and material type is significant. These results are consistent with our previous work and reports by other groups demonstrating that coupon surface characteristics are a significant factor with respect to RE [11, 13, 20].

The false negative rates (FNR) and limit of detection (LOD_90_) were calculated from the results of the experiments conducted with clean and grime coated coupons. Ceramic tile and stainless steel coupons had lower FNR and LOD values compared to vinyl tile, which is consistent with the results reported by Krauter et al. showing that reduced surface roughness had roughly linear dependency with spore recovery [10]. We also found that FNR and LOD_90_ were consistently lower with the MM-MPC method or MP-Discrete method compared to the SM-MPC method when four or eight samples were composited (Figs 9 and 10).

In conclusion, we have tested three composite sampling approaches at low CFU targets with four different coupon materials. Our results demonstrate that the multi-media multi-pass composite sampling (MM-MPC) methodology is a more effective sampling campaign at low spore concentrations compared to other composite sampling strategies tested. The RE for MM-MPC is higher than for the SP-Discrete, SM-SPC, and SM-MPC methods (Table 2, Figs 4 and 5), and has similar RE to a comparable discrete sampling method (MP-Discrete, Fig 5). Using this MM-MPC method to composite up to 16 sample locations could streamline laboratory analysis without sacrificing confidence in RE or increasing the FNR compared with the discrete sampling method (Figs 7, 9 and 10). The advantages of the MM-MPC method are reduced time, labor, and material requirements as described above. This approach could improve response times to biological events, reduce costs and labor requirements, and is an excellent candidate method for further testing and verification by other laboratories.

## Supporting Information

S1 Fig Plot of theoretical versus sample quantiles of the residuals shows the data has a normal distribution. (TIF)

S1 Table Recovery efficiency using the SM-SPC method versus the SM-MPC method at 100 CFU/coupon. (XLSX)

S2 Table Test Matrices for the full factorial composite study. (XLSX)

S3 Table Results from the full factorial study. (XLSX)

S4 Table Description of each experimental run using grime coated coupons with results. (XLSX)
