# Supplementary figures and images for: Evaluating Composite Sampling Methods of Bacillus Spores at Low Concentrations
Source: PLoS One. 2016 Oct 13;11(10):e0164582. doi: 10.1371/journal.pone.0164582 (PMC5063342; doi:10.1371/journal.pone.0164582)

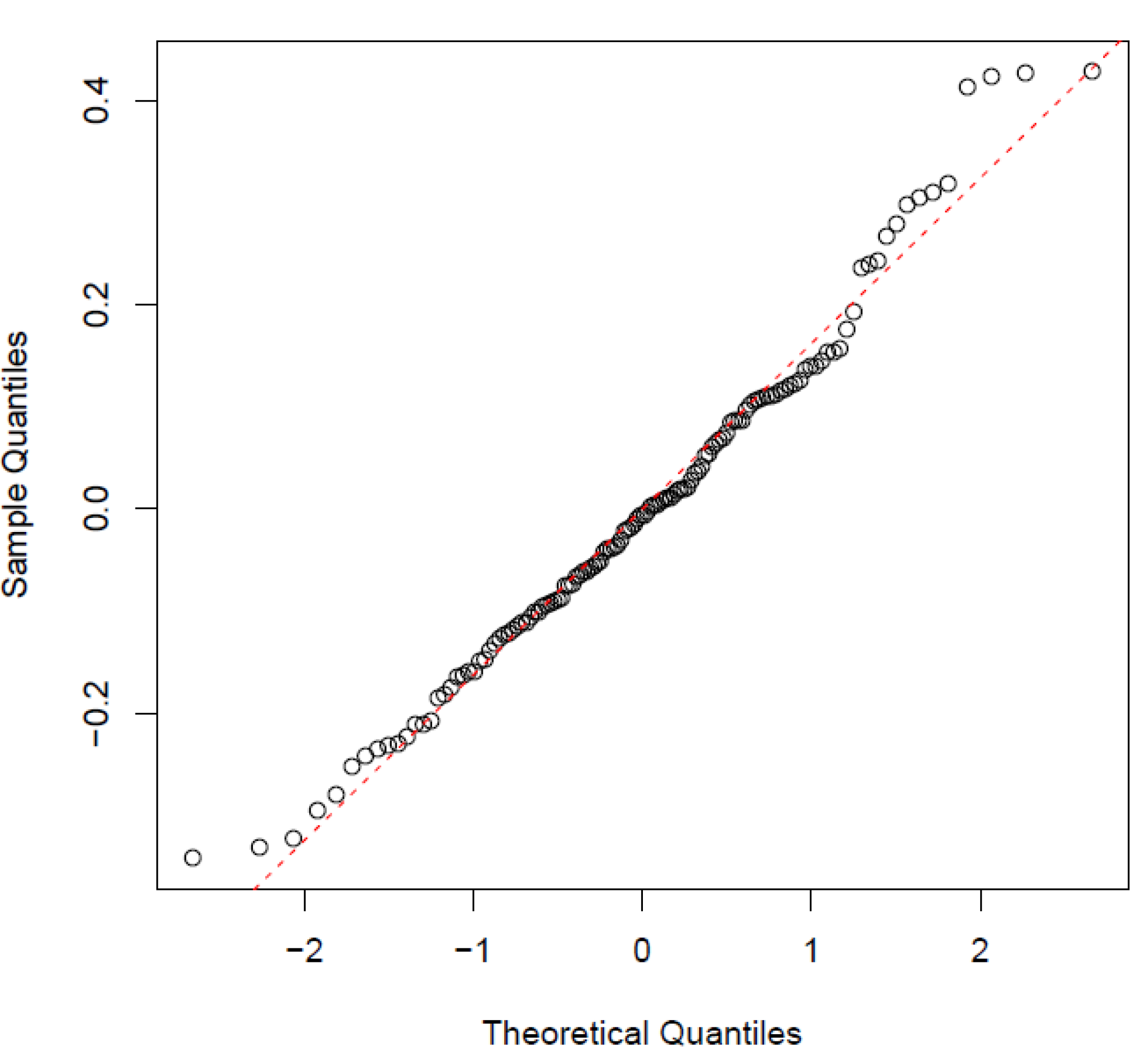

Supplement: S1 Fig — (TIF) [file pone.0164582.s001.tif]
